# Association between severe lumbar disc degeneration and self‐reported occupational physical loading

**DOI:** 10.1002/1348-9585.12316

**Published:** 2022-01-27

**Authors:** Sami Salo, Heidi Hurri, Toni Rikkonen, Reijo Sund, Heikki Kröger, Joonas Sirola

**Affiliations:** ^1^ Kuopio musculoskeletal research unit (KMRU), Surgery, Institute of Clinical Medicine University of Eastern Finland (UEF) Kuopio Finland; ^2^ Department of Orthopaedics, Traumatology and Hand Surgery Kuopio University Hospital Kuopio Finland

**Keywords:** intervertebral disc degeneration, lumbosacral region, occupational exposure, postmenopause, spine, women's health

## Abstract

**Objectives:**

Occupational physical loading has been reported to be associated with intervertebral disc degeneration. However, previous literature reports inconsistent results for different vertebral levels. The aim of our study was to investigate the association between lumbar disc degeneration (LDD) at different vertebral levels and the self‐reported physical loading of occupation.

**Methods:**

The study population consisted of 1,022 postmenopausal women and was based on the prospective Kuopio Osteoporosis Risk Factor and Prevention (OSTPRE) study cohort. The severity of LDD was graded from T2‐weighted MRI images using the five‐grade Pfirrmann classification. Five intervertebral levels (L1–L2 to L5–S1) were studied (total 5110 discs). The self‐rated occupational physical loading contained four groups: sedentary, light, moderate, and heavy.

**Results:**

The heavy occupational physical loading group had higher odds for severe LDD at the L5–S1 vertebral level (OR 1.86, 95% CI: 1.19–2.92, *p *= .006) in comparison with the sedentary work group. A clear trend of increasing disc degeneration with heavier occupational loading was also observed at the L5–S1 level. Age, smoking, and higher body mass index (BMI) were associated with more severe LDD. Leisure‐time physical activity at the age of 11–17 years was associated with less severe LDD. Controlling for confounding factors did not alter the results.

**Conclusions:**

There appears to be an association between occupational physical loading and severe disc degeneration at the lower lumbar spine in postmenopausal women. Individuals in occupations with heavy physical loading may have an increased risk for work‐related disability due to more severe disc degeneration.

## 1 INTRODUCTION

Low back pain is nowadays the leading cause of disability worldwide.^1^ Intervertebral disc degeneration has been found to be associated with low back pain^2^, ^3^, ^4^, ^5^, ^6^ and recurrent episodes of low back pain.^7^ However, disc degeneration does not always cause low back pain, and degenerative changes in the spine are highly common also among asymptomatic individuals.^8^, ^9^, ^10^ The severity of disc degeneration increases with age.^10^, ^11^

Occupational physical loading has been reported to be associated with disc degeneration.^12^, ^13^ However, mechanical loading factors may affect disc degeneration in various ways at different vertebral levels. Occupational lifting has been found to be associated significantly with disc degeneration at the L1–L2 level, but not at other lumbar vertebral levels.^14^ Work‐related mechanical lumbar loading may accelerate disc degeneration at the level L4–L5.^15^ Prolonged sitting, twisting/bending, lifting heavy objects, and heavy physical load were significantly associated with disc degeneration.^16^ Lumbar spondylosis was found to be significantly higher among agricultural, forestry, and fishery workers than among clerical workers and technical experts in the overall population.^17^ However, in a twin study, it was found that male occupational drivers did not have more severe disc degeneration than their twin brothers.^18^

A strong dose‐response relationship between cumulative lumbar load and lumbar disc herniation and also disc narrowing has been observed among both men and women.^19^ Occupational lifting by airport baggage handlers has been found to predict hospital admission due to low back pain, but no difference in the disc herniation rate was observed in comparison with a large reference group.^20^

A systematic review and meta‐analysis concluded moderate evidence of an association between occupational loading and disc degeneration in terms of signal intensity.^21^ Low‐quality‐grade evidence has been found between loading and disc height, with inconsistent results for different intervertebral levels. Low‐quality‐grade evidence of an association between occupational loading and disc bulging, Modic changes, osteophytes, Schmorl's nodes, and other endplate abnormalities have also been found.^21^

The aim of the present study was to investigate the association between occupational physical loading and lumbar disc degeneration in Finnish postmenopausal women using a five‐grade Pfirrmann disc degeneration classification system.^22^

## 2 MATERIALS AND METHODS

### 2.1 Study population

The study population was based on the prospective Kuopio Osteoporosis Risk Factor and Prevention (OSTPRE) study cohort. The protocol of the study has been described previously.^23^, ^24^

**FIGURE 1:**
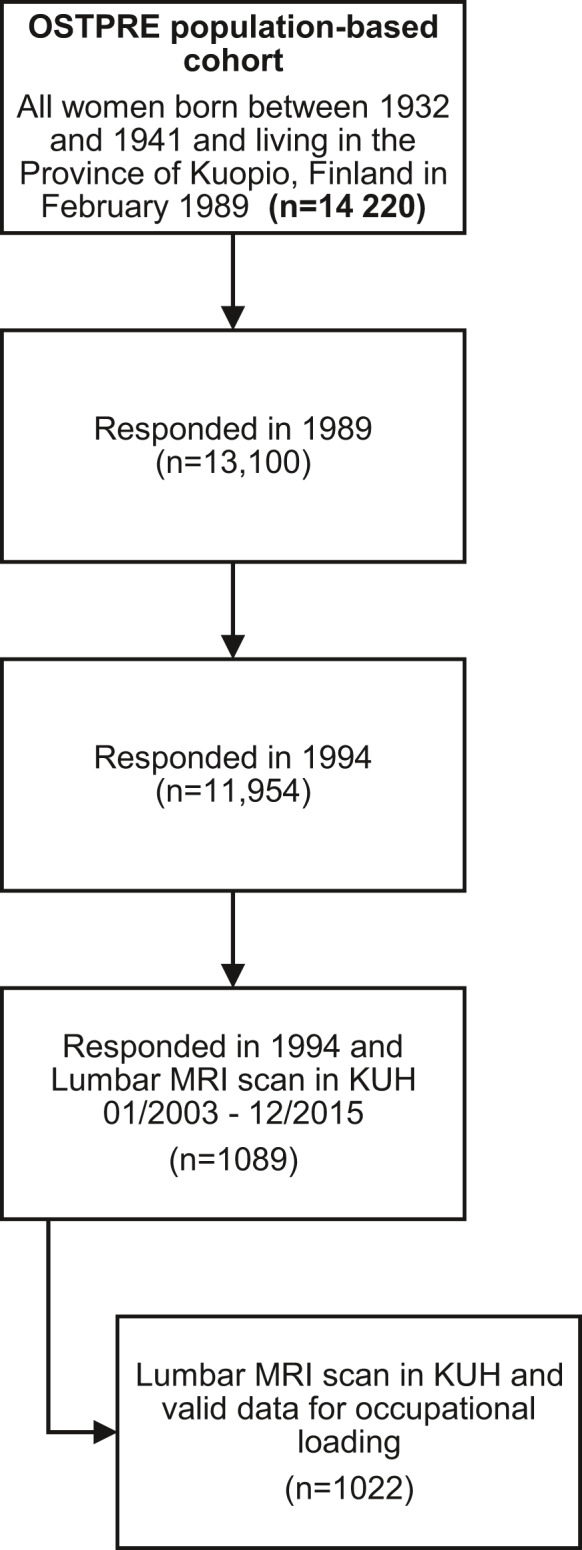
Selection of the study population

The selection of the study population is presented in Figure 1. The OSTPRE cohort was established in February 1989 by selecting all women born between 1932 and 1941 living in the Province of Kuopio (latitude 62–64°N) in Eastern Finland (*N* = 14 220). A self‐administered baseline questionnaire was mailed to a total of 14 220 participants in 1989, and a total of 13 100 women responded to the questionnaire. The baseline questionnaire included questions about health‐related factors, comorbidity, medications, and anthropometric measures. At the 5‐year follow‐up in 1994, a similar questionnaire was mailed to the 12 831 women, who had responded to the baseline enquiry (*N* = 13 100) and were alive at the time. The response was obtained from 11 954 women at the 5‐year follow‐up questionnaire. The response rate varied between 91% and 93%. Questions about the self‐rated physical loading of the respondent's most recent occupation were asked in the 5‐year follow‐up enquiry. Occupational physical loading was self‐rated and the classification in the 5‐year follow‐up enquiry contained four groups: (1) sedentary, (2) light, (3) moderate, and (4) heavy occupational physical loading. This classification system has been used by the Finnish Institute of Occupational Health while making workplace temperature recommendations according to different occupational physical loading levels. These recommendations and classification originate from the Finnish National Board of Health recommendations from the year 1978, which are based on i.a. ISO 7730 and ASHRAE 55–81 standards.

The occupational classification used in the present study is based on Finnish Occupational Classification 1980.^25^ This classification is based on the Nordic Occupational classification from the year 1963, which was made according to the International Standard Classification of Occupations (ISCO‐58) published in the year 1958 by the International Labour Organization (ILO).^26^ Finnish Job Exposure Matrix (FINJEM) data was used to evaluate the validity of the used occupational classification system. Development and validation for this matrix have been described previously.^27^ The best compatible occupational class from FINJEM‐database was selected for comparison to the occupational classes of the present study. Some occupational classes, which were too wide, were split into more specific subclasses. FINJEM‐database provides information about how large proportion of the workers in specific occupations are exposed to specific work‐load factors. Occupation‐specific work‐load factors from FINJEM‐database for two following occupational loading factors were used: (1) heavy physical work, (2) sitting. From the present study population, the proportion of the study subjects who had rated their work sedentary or heavy were compared to FINJEM exposure proportions.

During the follow‐up period (01/2003 to 12/2015) 1089 out of 11 954 5‐year follow‐up respondents had a lumbar MRI scan in Kuopio University Hospital (KUH). A valid response to the occupational loading question and valid data on height, weight, medical conditions, and smoking history were obtained in total for 1042 women. This data was based on the 5‐year follow‐up questionnaire. However, 20 out of the 1042 women had missing information for a specific occupation class and these 20 women were excluded from the final study sample. Hence the final study sample consisted of 1022 women. Valid data for these same variables, except lumbar MRI were obtained from 9973 women in the whole OSTPRE study cohort. This group was used as a reference group in order to investigate the differences in characteristics between women who had a lumbar MRI scan and those who did not have a lumbar MRI scan at KUH. In addition, the 5‐year questionnaire included questions about leisure time physical activity (average hours per week) during the last year, leisure time physical activity at the age of 11–17 years (Yes/No), and years worked in the occupation. Valid data for all these variables were obtained in total from 892 women.

The study protocol was approved by the ethics committee of Kuopio University Hospital (KUH). Diagnosis or indication related to lumbar MRI was obtained from Care Register for Health Care (CRHC). Ostpre study has approval for use of this register data. Informed written consent from the participants was collected.

### 2.2 Magnetic resonance imaging

The MRI scans of the lumbar spine were performed with a 1.5 T MRI scan unit. The images were obtained from the KUH image database PACS (Picture Archiving and Communication System, Sectra), which has been available since 2002. The scans were performed between 01/2003 and 12/2015. Some of the women had several MRI scans in PACS. If so, the first MRI scan of each woman was used to evaluate the severity of disc degeneration. All MRI scans were performed due to any clinical indication for an MRI scan of the lumbar spine. These indications contained for example back pain (or back pain and earlier cancer diagnosis), neurological symptoms of the lower legs, and spinal claudication or stenosis symptoms etc.

### 2.3 Disc degeneration grade

The severity of intervertebral disc degeneration was graded at five vertebral levels from the L1–L2 level to the L5–S1 level. The evaluation was performed on T2‐weighted images using the 5‐grade disc degeneration classification system introduced by Pfirrmann et al.^22^: Grade 1: Normal disc height and shape, the distinction between nucleus and annulus is clear with a bright hyperintense signal of the nucleus. Grade 2: Normal disc height but nonhomogeneous structure of the disc, with a hyperintense white signal of the nucleus with or without horizontal grey bands. The distinction between annulus and nucleus is clear. Grade 3: Normal or slightly decreased disc height, nonhomogeneous structure of the disc with an intermediate grey signal intensity of the nucleus. The distinction between annulus and nucleus is unclear. Grade 4: Normal or moderately decreased disc height, nonhomogeneous structure of the disc with a hypointense dark grey signal intensity of the nucleus. The distinction between annulus and nucleus is impossible to observe. Grade 5: Collapsed disc space, nonhomogeneous structure of the disc with hypointense black signal intensity. The distinction between annulus and nucleus is lost. The intra‐^22^ and interobserver^22^, ^28^ reliability of this disc degeneration classification system has been reported to be from substantial to excellent.

The disc degeneration grade was evaluated prior to statistical analyses in order to blind the study group for occupational loading. These results were not altered after the acquisition of the disc degeneration grade.

### 2.4 Statistical analysis

All statistical analyses were carried out using SPSS, version 27.0.1.0. A mean degeneration grade of all five discs (L1–L2 to L5–S1) was calculated for each woman individually. The degeneration grade was divided into two groups at each vertebral level. The first group had degeneration grades 1 to 4 according to the Pfirrmann degeneration classification system. This was considered non‐severe degeneration, whereas degeneration grade 5 was considered severe degeneration. The mean degeneration grade was also divided into two groups. The first group had a mean degeneration grade less than 4, which was considered non‐severe degeneration. The second group had a mean degeneration grade from 4 to 5 which was considered as severe degeneration. Differences in the mean age between non‐severe and severe disc degeneration groups were analyzed using the independent samples T‐test. The Chi‐squared test was used to compare the distribution of severe and non‐severe disc degeneration between different occupational loading groups.

Differences between the MRI subsample group and reference group were analyzed using the independent samples T‐test for height, weight, body mass index (BMI), the total amount of medical conditions, average smoking history in years, leisure time physical activity (average hours per week) during the last year before 5‐year questionnaire, and working years in the occupation. The Chi‐squared test was used for categorical variables including occupational physical loading, ever‐smoking (Yes/No), and leisure time physical activity during the last year before the 5‐year questionnaire (Yes/No). Levene's test was used before all the T‐tests to test for the equality of variances. The difference in the mean degeneration grade between different age groups was investigated using ANOVA analysis. Additionally, the Pearson correlation coefficient was used while inspecting the association between mean degeneration and aging.

Binary logistic regression analysis was used to calculate odds ratios (OR) for severe degeneration. Sedentary work was used as the reference group. *p *< .05 was considered to be statistically significant. Age at the time of MRI scan, BMI, smoking history in years, time from the 5‐year follow‐up questionnaire to MRI scan, the total number of chronic medical conditions, leisure time physical activity during the last year before the 5‐year questionnaire (average hours per week), leisure time physical activity at the age of 11–17 years (Yes/No), and working years in the occupation were used as covariates in the analyses. These same covariates were used as covariates in ANCOVA analysis when investigating the difference in the mean degeneration grade between different age groups. The interaction between BMI and occupational physical loading was investigated by including the interaction term (BMI * physical loading) between these variables into logistic regression analyses.

## 3 RESULTS

Characteristics of the present study population are presented in Table 1. The mean age at the time of the MRI scan was 73.0 years (SD 4.4). Time from answering the questionnaire on occupational physical loading to the date of MRI scan was, on average, 15.9 years (SD 3.7). The sedentary work group involved 17.1% of the study population, representing the lightest occupational loading group. Physically light work was the smallest group, involving only 9.4% of the study population. Moderate occupational physical loading was the largest group, representing 42.1% of the study population. The rest of the participants, 31.4%, had rated the physical loading of their occupation as heavy. The mean degeneration grade of each intervertebral level, according to the Pfirrmann degeneration classification system, is also presented in Table 1. The distribution of the study population into different occupational classes according to occupational physical loading is presented in Table S1. In addition, the study population's occupational physical loading comparison to FINJEM data in different occupational classes is presented in Table S4.

**TABLE 1:** Characteristics of the study population

	MRI subsample *N* = 1022		Reference sample *N* = 9973	
	Mean (SD)	Range	Mean (SD)	*p*‐value
Age on the MRI date (years)	73.0 (4.4)	61.3–83.3		
Sedentary work group	73.0 (4.5)			
Light work group	72.8 (4.6)			
Moderate work group	72.8 (4.4)			
Heavy work group	73.2 (4.3)			
Height (cm)	161.9 (5.1)	147.0–178.0	161.2 (5.2)	<.001
Weight (kg)	70.8 (11.2)	42.0–158.0	70.4 (12.3)	.258
Body mass index (m2/kg)	27.0 (4.0)	18.1–44.6	27.1 (4.5)	.692
Medical conditions	1.8 (1.5)	0–10	1.5 (1.5)	<.001
Time from questionnaire to MRI (years)	15.9 (3.7)	8.6–21.6		
Ever‐smoker	201 (19.7%)		1815 (18.2%)	.248
Average smoking history, years	3.41 (8.5)	0.0–42.0	3.2 (8.3)	.341
Regular leisure time physical activity during last year^a^				.320
Yes	505 (48.2%)		4795 (49.9%)	
No	470 (51.8%)		4819 (50.1%)	
Leisure time physical activity during last year (average hours per week)^b^	2.5 (4.3)	0.0–46.0	2.4 (4.7)	.789
Leisure time physical activity at the age of 11–17 years^c^				.607
Yes	459 (47.8%)		4572 (48.6%)	
No	502 (52.2%)		4829 (51.4%)	
Distribution of occupational physical loading				.013
Sedentary work	175 (17.1%)		1572 (15.8%)	
Light work	96 (9.4%)		1138 (11.4%)	
Moderate work	430 (42.1%)		4499 (45.1%)	
Heavy work	321 (31.4%)		2764 (27.7%)	
Working years in the occupation^d^	24.2 (11.2)	0–55	24.8 (11.4)	.111
Disc degeneration grade (MRI subsample)				
L1–L2	3.40 (0.64)			
L2–L3	3.55 (0.66)			
L3–L4	3.58 (0.63)			
L4–L5	3.84 (0.64)			
L5–S1	3.92 (0.82)			
L1–S1 mean degeneration grade	3.66 (0.41)			
Age groups (MRI subsample)	*N* (%)			
Under 65 years	51 (5.0%)			
65–69.9 years	208 (20.4%)			
70–74.9 years	400 (39.1%)			
Over 80 years	42 (4.1%)			

Note.

Differences between the MRI subsample group and the reference group were analyzed using independent samples T‐test for height, weight, BMI, the total amount of medical conditions, average smoking history in years, leisure time physical activity during the last year before 5‐year questionnaire (average hours per week), and working years in the occupation. The Chi‐squared test was used for categorical variables including occupational physical loading, ever‐smoking (Yes/No), and leisure time physical activity (Yes/No).

^a^ Covariate was available for 975 in the MRI sample and 9614 in the reference sample.

^b^ Covariate was available for 960 in the MRI sample and 9415 in the reference sample.

^c^ Covariate was available for 961 in the MRI sample and 9401 in the reference sample.

^d^ Covariate was available for 998 in the MRI sample and 9681 in the reference sample.

Characteristics of the reference sample are also presented in Table 1. The average height was slightly higher in the MRI subsample group. The total number of chronic medical conditions was a bit higher in the MRI subsample group. The proportion of women in sedentary and heavy occupational loading groups was slightly higher in the MRI subsample group than in the reference sample group.

A clear trend of increasing mean degeneration grade along with age can be seen in Figure S1. The difference in the mean degeneration grade between the age groups was significant (*p *< .001). Vertical lines represent the 95% confidence intervals of the mean degeneration grade of all five lumbar vertebral levels (L1–S1) according to age groups. The increase in degeneration also slowed down with age.

The number of evaluated intervertebral discs for the study population (*N* = 1022) was 5110 (L1–L2 to L5–S1 vertebral levels). The distribution of disc degeneration at different vertebral levels is presented in Table S2. The majority of the discs (97.9%) were within the higher degeneration groups 3–5: Only three discs had Pfirrmann degeneration grade 1. There were 101 grade‐2 discs (2.0%), 2133 grade‐3 discs (41.7%), 2280 grade‐4 discs (44.6%), and 593 grade‐5 discs (11.6%) (Table S2). The severity of disc degeneration was more substantial at the two lowest lumbar vertebral levels (Table 1 and Table S2).

The proportion of severe degeneration (Pfirrmann degeneration grade 5) at different vertebral levels and for different occupational loading groups can be seen in Table 2. The proportion of severe disc degeneration was clearly more substantial at the lowest two lumbar vertebral levels. The proportion of severe disc degeneration at the L5–S1 intervertebral level was 25.5%. The proportion of severe degeneration at the L5–S1 level was higher for the heavy occupational loading group 97/321 (30.2%) than for the other groups. When observing the mean degeneration grade of all five discs (L1–S1) the proportion of the severe degeneration for the heavy occupational group was 95/321 (29.6%), which was also higher than for the other groups. The distribution of severe and non‐severe disc degeneration between occupational physical loading groups at different vertebral levels differed statistically significantly only at the L5–S1 level (*p *= .037). The mean age was higher for most of the severe degeneration groups compared to the non‐severe degeneration groups. *p*‐values from the independent samples T‐test, comparing differences of the mean ages between the groups, are presented in Table 2. The distribution of diagnosis or indication related to lumbar MRI is presented in Table S3. Also, the mean degeneration grade for the whole lumbar spine as well as the proportion of severe mean degeneration for each diagnosis group is presented in Table S3. Spinal stenosis was the largest diagnosis group covering 45.4% of all MRI scans.

**TABLE 2:** Proportion of severe degeneration at different vertebral levels and in different occupational loading groups

Vertebral level	Non‐severe degeneration	Non‐severe group mean age, years (SD)	Severe degeneration	Severe group mean age, years (SD)	*p*‐value
L1–L2	972 (95.1%)	72.9 (4.4)	50 (4.9%)	73.6 (4.6)	.304
L2–L3	941 (92.1%)	72.9 (4.5)	81 (7.9%)	73.9 (4.0)	.044
L3–L4	954 (93.3%)	72.9 (4.4)	68 (6,7%)	73.4 (4.3)	.385
L4–L5	889 (87.0%)	72.8 (4.6)	133 (13.0%)	73.8 (4.1)	.016
L5–S1	761 (74.5%)	72.8 (4.6)	261 (25.5%)	73.5 (4.0)	.020
Total discs	4517 (88.4%)		593 (11.6%)		
L1–S1 mean degeneration	751 (73.5%)	72.7 (4.6)	271 (26.5%)	73.8 (3.9)	<.001
Sedentary work (*n* = 175)					
L1–L2	167 (95.4%)	72.9 (4.5)	8 (4.6%)	74.9 (5.3)	.219
L2–L3	166 (94.9%)	72.8 (4.5)	9 (5.1%)	76.2 (2.6)	.004
L3–L4	165 (94.3%)	72.7 (4.5)	10 (5.7%)	77.0 (2.2)	<.001
L4–L5	154 (88.0%)	72.9 (4.6)	21 (12.0%)	73.4 (4.0)	.660
L5–S1*	145 (81.5%)	72.9 (4.7)	33 (18.5%)	73.3 (3.8)	.583
L1–S1 mean deg.	132 (75.4%)	72.5 (4.6)	43 (24.6%)	74.4 (3.9)	.023
Light work (*n* = 96)					
L1–L2	92 (95.8%)	72.6 (4.6)	4 (4.2%)	77.5 (1.1)	<.001
L2–L3	91 (94.8%)	72.7 (4.7)	5 (5.2%)	75.3 (2.8)	.216
L3–L4	91 (94.8%)	72.8 (4.7)	5 (5.2%)	72.6 (4.2)	.913
L4–L5	85 (88.5%)	72.8 (4.7)	11 (11.5%)	73.1 (4.4)	.858
L5–S1*	78 (78.8%)	72.2 (4.8)	21 (21.2%)	75.1 (3.0)	.001
L1–S1 mean deg.	72 (75.0%)	72.3 (4.9)	24 (25.0%)	74.4 (3.2)	.018
Moderate work (*n* = 430)					
L1–L2	407 (94.7%)	72.8 (4.5)	23 (5.3%)	74.0 (3.7)	.211
L2–L3	391 (90.9%)	72.7 (4.5)	39 (9.1%)	74.7 (2.7)	<.001
L3–L4	399 (92.8%)	72.9 (4.5)	31 (7.2%)	72.1 (4.0)	.332
L4–L5	374 (87.0%)	72.7 (4.4)	56 (13.0%)	73.6 (4.4)	.146
L5–S1*	320 (74.4%)	72.6 (4.6)	110 (25.6%)	73.5 (3.9)	.099
L1–S1 mean deg.	321 (74.7%)	72.4 (4.6)	109 (25.3%)	74.0 (3.7)	<.001
Heavy work (*n* = 321)					
L1–L2	306 (95.3%)	73.3 (4.3)	15 (4.7%)	71.3 (5.1)	.087
L2–L3	293 (91.3%)	73.3 (4.3)	28 (8.7%)	71.9 (5.1)	.109
L3–L4	299 (93.1%)	73.1 (4.3)	22 (6.9%)	73.8 (4.7)	.480
L4–L5	276 (86.0%)	73.0 (4.4)	45 (14.0%)	74.4 (3.7)	.019
L5–S1*	224 (69.8%)	73.1 (4.4)	97 (30.2%)	73.2 (4.2)	.854
L1–S1 mean deg.	226 (70.4%)	73.1 (4.3)	95 (29.6%)	73.2 (4.4)	.868

Note.

Differences in the mean age between non‐severe and severe disc degeneration groups were analyzed using independent samples T‐test. *p*‐values from the T‐test are presented in the table.

* *p* = .037, Chi‐squared test was used to compare the distribution of severe and non‐severe disc degeneration between different occupational loading groups.

**FIGURE 2:**
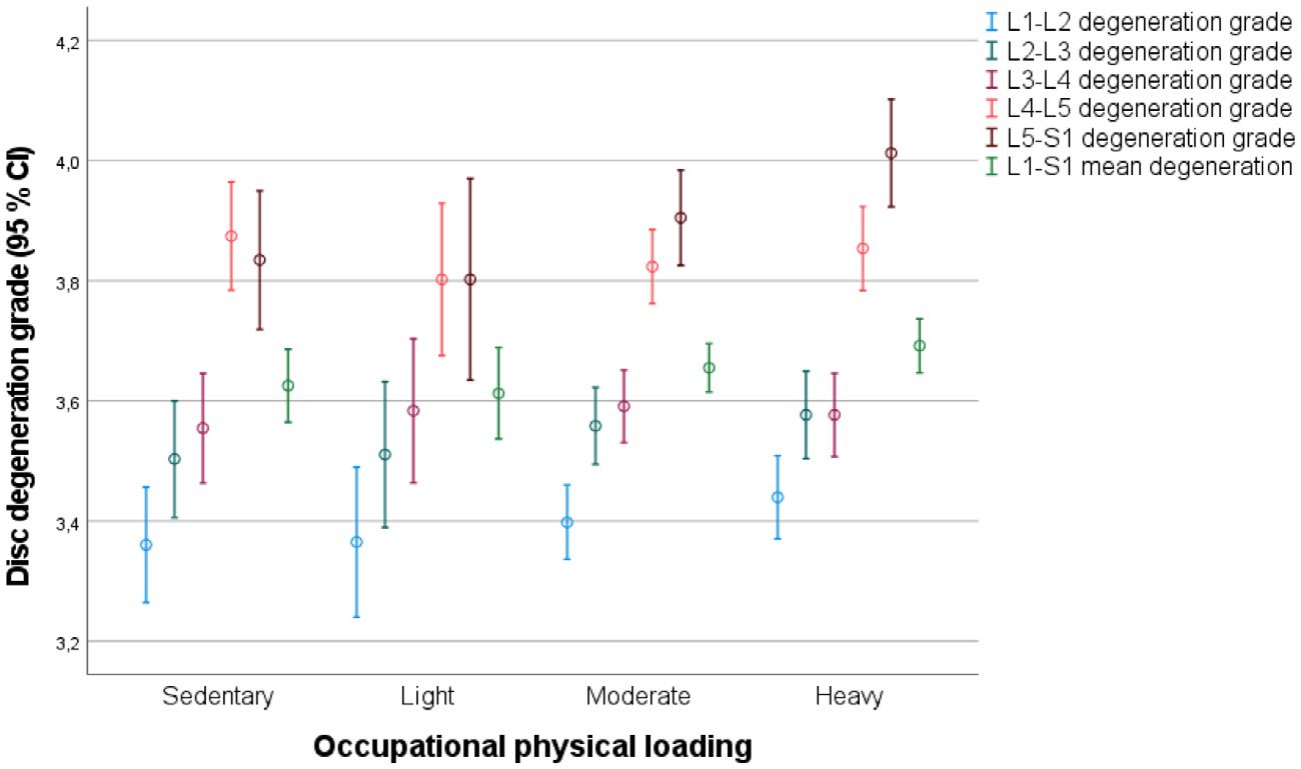
Occupational physical loading and disc degeneration

A clear trend of increasing disc degeneration grade with movement in the caudal direction can be seen in Figure 2. Vertical lines represent 95% confidence intervals of the disc degeneration grade at different vertebral levels, according to self‐rated occupational physical loading. It can be seen clearly in the figure that the lowest two lumbar vertebral levels had significantly more severe disc degeneration. A slight increase in the mean degeneration grade along with increasing occupational loading was found for most vertebral levels when comparing different occupational physical loading groups.

The heavy occupational physical loading group had higher odds for severe disc degeneration at the L5–S1 lumbar vertebral level than the sedentary work group (OR 1.86, 95% CI: 1.19–2.92, *p *= .006). Higher odds for severe degeneration were also found in a comparison of the moderate physical loading group with the sedentary group but the result did not have statistical significance (OR 1.48, 95% CI: 0.96–2.29, *p *= .079). A clear trend of ascending odds ratio with increasing occupational loading was observed at the L5–S1 level. Some similar trends were also found at the L2–L3 and L4–L5 levels, and at the L1–S1 mean degeneration grade of all five studied intervertebral levels. However, these results were statistically insignificant. Controlling for several confounding factors in the adjusted logistic regression model did not alter the results. The results of binary logistic regression analyses are presented in Table 3.

**TABLE 3:** Odds ratios (OR) for severe disc degeneration vs. non‐severe degeneration for different occupational loading groups and vertebral levels: logistic regression analysis

	OR	95% CI	*p*‐value	Adjusted OR	Adjusted *p*‐value
L1–L2 severe degeneration					
Light	0.91	0.27–3.10	0.877	0.67	.571
Moderate	1.18	0.52–2.69	0.694	1.02	.970
Heavy	1.02	0.43–2.46	0.959	0.94	.899
L2–L3 severe degeneration					
Light	1.01	0.33–3.11	0.981	1.22	.743
Moderate	1.84	0.87–3.88	0.110	2.05	.080
Heavy	1.76	0.81–3.83	0.152	1.98	.110
L3–L4 severe degeneration					
Light	0.91	0.30–2.73	0.862	0.94	.914
Moderate	1.28	0.61–2.68	0.508	1.18	.670
Heavy	1.21	0.56–2.63	0.622	1.23	.611
L4–L5 severe degeneration					
Light	0.95	0.44–2.06	0.895	0.93	.856
Moderate	1.10	0.64–1.88	0.732	1.17	.596
Heavy	1.20	0.69–2.08	0.527	1.16	.634
L5–S1 severe degeneration					
Light	1.21	0.65–2.23	0.552	1.27	.469
Moderate	1.48	0.96–2.29	0.079	1.50	.089
Heavy*	1.86*	1.19–2.92*	0.006*	1.79*	.018*
L1–S1 mean degeneration grade, severe degeneration					
Light	1.02	0.58–1.82	0.938	0.87	.672
Moderate	1.04	0.69–1.57	0.842	1.02	.947
Heavy	1.29	0.85–1.96	0.233	1.23	.382

Note.

The lightest occupational loading group, sedentary work, was used as the reference group.

The model was adjusted for body mass index (BMI), age at the time of MRI scan, smoking history in years, time from 5‐year follow‐up questionnaire to MRI scan, the total number of chronic medical conditions, leisure time physical activity during last year before the 5‐year questionnaire (average hours per week), leisure time physical activity at the age of 11–17 years (Yes/No) and working years in the occupation.

In the un‐adjusted model *N* was 1022 and in the adjusted model *N* was 892.

* Significance (*p* < .05).

When several confounding factors were taken into account as covariates in an adjusted logistic regression model, the following covariates were statistically significant in the following analyses: Age at the time of MRI scan was a significant covariate for the L1–S1 mean degeneration grade analysis. BMI was a significant covariate for the L1–L2 level and for the L1–S1 mean degeneration grade analysis. Smoking history in years was significant for the L4–L5 level analysis. Leisure time physical activity at the age of 11–17 years was a significant covariate for L2–L3 level and for the L1–S1 mean degeneration grade analysis. Age, smoking, and BMI did increase the odds for severe degeneration. Leisure‐time physical activity at the age of 11–17 years did decrease the odds for severe degeneration. Time from 5‐year follow‐up questionnaire to MRI scan, the total number of medical conditions, leisure time physical activity (average hours per week), and working years in the occupation were not significant covariates in any of the analyses. Interaction between BMI and occupational physical loading was not significant for any of the studied vertebral levels. All odds ratios of the covariates in the adjusted logistic regression model are presented in Table 4.

**TABLE 4:** Odds ratios of the covariates in the adjusted logistic regression model presented in Table 3

Covariate	OR	95% CI	*p*‐value
L1–L2			
BMI*	1.08*	1.01–1.16*	.031*
Age	1.02	0.91–1.15	.692
Time from questionnaire to MRI	1.05	0.92–1.20	.502
Smoking history in years	1.01	0.98–1.05	.427
Number of medical conditions	0.97	0.78–1.21	.782
Leisure time physical activity (average hours per week)	0.93	0.83–1.04	.188
Leisure time physical activity at the age of 11–17 years	0.56	0.29–1.06	.073
Working years in the occupation	0.99	0.96–1.02	.489
L2–L3			
BMI	1.01	0.96–1.08	.655
Age	1.05	0.96–1.14	.334
Time from questionnaire to MRI	1.04	0.93–1.16	.525
Smoking history in years	1.01	0.98–1.04	.432
Number of medical conditions	1.10	0.93–1.30	.248
Leisure time physical activity (average hours per week)	0.94	0.86–1.02	.125
Leisure time physical activity at the age of 11–17 years*	0.58*	0.35–0.96*	.034*
Working years in the occupation	1.01	0.99–1.03	.329
L3–L4			
BMI	1.01	0.95–1.08	.695
Age	1.01	0.92–1.11	.863
Time from questionnaire to MRI	1.02	0.91–1.15	.691
Smoking history in years	1.01	0.98–1.04	.521
Number of medical conditions	0.94	0.77–1.14	.540
Leisure time physical activity (average hours per week)	1.02	0.96–1.08	.575
Leisure time physical activity at the age of 11–17 years	0.85	0.50–1.45	.549
Working years in the occupation	1.01	0.98–1.03	.518
L4–L5			
BMI	0.99	0.94–1.04	.713
Age	1.05	0.98–1.13	.179
Time from questionnaire to MRI	1.03	0.94–1.12	.537
Smoking history in years*	1.02*	1.00–1.04*	.045*
Number of medical conditions	1.12	0.98–1.28	.086
Leisure time physical activity (average hours per week)	1.03	0.99–1.07	.147
Leisure time physical activity at the age of 11–17 years	1.02	0.69–1.51	.936
Working years in the occupation	0.99	0.98–1.01	.513
L5–S1			
BMI	1.02	0.98–1.06	.251
Age	1.05	0.99–1.11	.121
Time from questionnaire to MRI	0.99	0.92–1.05	.655
Smoking history in years	1.00	0.98–1.02	.750
Number of medical conditions	1.05	0.94–1.17	.383
Leisure time physical activity (average hours per week)	0.97	0.93–1.02	.205
Leisure time physical activity at the age of 11–17 years	0.97	0.72–1.33	.891
Working years in the occupation	1.00	0.99–1.02	.704
L1–S1 mean degeneration grade			
BMI*	1.04*	1.00–1.09*	.032*
Age*	1.07*	1.01–1.13*	.020*
Time from questionnaire to MRI	1.01	0.94–1.08	0.892
Smoking history in years	1.01	0.99–1.03	0.217
Number of medical conditions	1.00	0.89–1.11	0.943
Leisure time physical activity (average hours per week)	0.98	0.94–1.02	0.391
Leisure time physical activity at the age of 11–17 years*	0.70*	0.51–0.95*	.021*
Working years in the occupation	1.00	0.98–1.01	0.781

Note.

* Significance (*p* < .05).

## 4 DISCUSSION

The present cross‐sectional population‐based study investigated the association between occupational physical loading and the severity of intervertebral disc degeneration in the lumbar spine. It was found that higher occupational physical loading was related to more severe disc degeneration. The result was significant at the lowest lumbar vertebral level, between the L5‐ and S1 vertebras. The result remained significant after controlling for several confounding factors, including age, BMI, time from occupational loading questionnaire to MRI scan, smoking history in years, the total number of chronic medical conditions, leisure time physical activity in middle age, leisure time physical activity at adolescence, and working years in the occupation. Age was a significant covariate, and the severity of disc degeneration increased along with aging. However, a trend of ascending degeneration grade along with an increase in occupational physical loading was found for most vertebral levels.

Some previous studies have found that the association between occupational physical loading and intervertebral disc degeneration varies at different vertebral levels.^14^, ^15^, ^29^ A systematic review and meta‐analysis have concluded moderate evidence of an association between occupational loading and disc degeneration in terms of signal intensity. Depending on the number and quality of the studies included in the review, the evidence was classified into high‐, moderate‐, low‐, or very low‐quality evidence. Low‐quality grade evidence was found between loading and disc height with inconsistent results between different intervertebral levels.^21^ Our study combined signal intensity decrease and intervertebral disc height reduction with the MRI‐based classification system introduced by Pfirrmann et al.^22^ Our results are in accordance with these previous studies, as our findings for the association between occupational physical loading and severe disc degeneration were significant only at the L5–S1 vertebral level and not at the other lumbar vertebral levels. However, a clear trend between greater occupational physical loading and more severe disc degeneration was found at most of the vertebral levels. A lower proportion of severe degeneration at the upper lumbar spine level may be one reason why our findings for the association between occupational physical loading and severe disc degeneration were statistically insignificant at the upper lumbar spine level.

Age, BMI, smoking history in years, and leisure time physical activity at the age of 11–17 years were significant covariates in some of the analyses. (Table 4) However, their effect on findings was rather minor. Aging is known to be one of the most important risk factors for disc degeneration.^11^, ^30^ The mean age was higher in most of the severe‐degeneration groups. (Table 2) Most of our study subjects were aged between 65 and 80 years. The under 65 years group and over 80 years group were rather small, and 95% confidence intervals were quite wide in these groups because of their small sample size. As a clear increase in disc degeneration along with aging was observed, the results of the present study support the connection between aging and disc degeneration. (Figure S1) High body mass index (BMI) has been associated with disc degeneration.^31^ This association may be distinct in different ethnicities. Higher BMI was found to be associated with excess risk for lumbar degenerative disease in a British population but not in a Japanese population.^32^ However, the structures of the spine including intervertebral discs adapt to loading, and a higher body mass index may not always be harmful to intervertebral discs.^33^ It has been suggested that greater routine physical loading may even be beneficial for intervertebral discs.^33^, ^34^ A lack of sports activities has been found to be a risk factor for the development of lumbar disc degeneration.^35^ The molecular changes underlying the harmful effects of aging, smoking, and obesity were recently investigated in a comprehensive review.^36^ Smoking has been found to have consistent associations with disc degeneration and low back pain in epidemiological studies.^37^ Cell‐level harmful effects of smoking on disc degeneration have also been found.^38^ These findings in the literature may explain why these covariates appeared to be significant in the present study. Although the odds ratio for BMI was comparatively small, the units of measurement for BMI and the degeneration grade should be taken into account when interpreting results, as BMI ranged from 18.1 to 44.6 (mean 27.0, SD 4.0), but disc degeneration grade ranged between 1 and 5 (mean 3.40–3.92, SD 0.41–0.82). (Table 1) Results of the present study also suggest an association between higher BMI and more severe disc degeneration in the lumbar spine. Leisure time physical activity at the age of 11–17 years appeared to be a significant covariate in L2–L3 level analysis (OR 0.58, 95% CI: 0.35–0.96, *p *= .034), as well as L1–S1 mean degeneration grade analysis (OR 0.70, 95% CI: 0.51–0.95, *p *= .021). In addition, odds ratios below 1.0 were observed also at all other analyses except the L4–L5 level analysis. However, these results were statistically insignificant. Odds ratios of all used cos can be found in Table 4. These results may indicate that leisure time physical activity in adolescence may have some preventive effects against severe intervertebral disc degeneration at the lumbar spine. This possible preventive factor should be studied more precisely in further studies.

Average height, the total number of chronic medical conditions, and the distribution of occupational physical loading differed between the MRI subsample and the whole OSTPRE study cohort reference group. Although these differences were statistically significant, they were still relatively small. A slightly higher proportion of the study subjects from the sedentary and the heavy occupational loading group had a lumbar MRI scan during the follow‐up. This may indicate that heavy occupational loading, but also sedentary work may increase the risk for clinical back problems. Reasonable loading in light and moderate occupational loading groups may even have some beneficial and preventive effects on clinical back problems. In addition, the light occupational physical loading groups had lower odds ratios for severe degeneration compared to the sedentary work groups at the L1–L2, L3–L4, and L4–L5 level analysis. It is possible that light occupational physical loading may have some preventive effects against severe disc degeneration compared to sedentary work. Prolonged sitting has been found to be significantly associated with lumbar disc degeneration.^16^ This may give some explanation for findings in the present study. However, subsequent of these results, from the logistic regression analyses were statistically insignificant and should be interpreted with caution.

The strengths of the present study include the large population‐based study sample including an entire age cohort. The original OSTPRE cohort was established in February 1989 by selecting all women born between 1932 and 1941 and living in the Province of Kuopio (latitude 62–64°N) in Eastern Finland (*N* = 14 220). The majority of the study population had a long career in the answered occupation (mean 24.2 years, SD 11.2). Additionally, the length and variation of the working years in the occupation were rather similar in the MRI sample compared to the reference sample. To the best of our knowledge, the present study had the largest sample size of the published studies so far that investigated the association between occupational loading and intervertebral disc degeneration evaluated from MRI images. Several important confounding factors were used as covariates in the analyses, which did not alter the results. MRI scans were performed by trained personnel, and quality standards were high. Evaluation of the disc degeneration grade was performed blinded to occupational physical loading data prior to analyses. While interpreting the differences between the MRI subsample and reference groups, it was concluded that the MRI group was a rather representative subsample of the original OSTPRE study cohort.

There are some limitations to the framework and methodology of the present study. The final study population of 1022 women represented only a small part of the original OSTPRE study cohort. The study population consisted only of women. The time difference between MRI scans and information from self‐reported occupational physical loading from the most recent occupation was rather long, however, this time difference was used as a covariate in the analyses, and it was not statistically significant. The occupational physical loading was only self‐rated, and this may lead to an overstatement of the results of the present study. The study subjects physical condition, health status, and also job satisfaction may have affected the self‐rating of occupational physical loading. It is possible that some of the study subjects, even those in sedentary work, have partially rated their work physically heavy because they already had lumbar disc degeneration and probably some symptoms from it. However, the occupational physical loading may also variate a lot inside the same occupational class depending on the workplace and also the specific occupation inside the same class. For some largest occupational classes, a comparison to FINJEM data was done (Table S4). The results were quite similar between the present study population and FINJEM data. For example, 83.33% of “Secretaries and typists” had rated their work sedentary in the present study sample, whereas according to FINJEM data, 91.14% of Secretaries are exposed to “sitting”. In the present study sample, 48.14% of the “building caretakers and cleaners” had rated their work as heavy, while in the FINJEM data, 54.12% of “cleaners” are considered to be exposed to “heavy physical work.” Also in many other classes, the results were rather similar. The largest occupation subclass in the present study sample was “Farmer's wife,” as 149 women had answered this as their occupation. There was not a corresponding occupational subclass in the FINJEM data for this occupation. Occupation contains traditionally a lot of farmwork, but also a lot of housekeeping, cooking, and childcare work. During the working careers of the study sample, which mainly date from the 1950s to the 1990s, this was a common occupation in the Finnish countryside. Additionally, the occupational physical loading may variate a lot in agricultural work depending on the field of agriculture (dairy farm, cereal farming, horticulture etc.) and also the size of the farm. There were also occupational classes that differed between the study sample and FINJEM‐data. However, the occupational classification used in the present study and the FINJEM‐data are a bit different, as FINJEM data is based on a newer occupational classification, and also the offset for these two classifications differs from each other.

MRI scans were performed due to clinical indications of lumbar MRI, and this may have affected the results. Thus, the individuals with clinical lower back problems were clearly overrepresented in the study sample. The distribution of diagnosis or indication related to lumbar MRI is presented in Table S3. Spinal stenosis was the largest diagnosis group covering 45.4% of all MRI scans. The average degeneration grade would have likely been less severe for a random sample of the OSTPRE study population, and this may have affected also the results of the study. However, it is difficult to evaluate the direction and significance of this possible effect. Valid data on the duration of the continuous working postures or other information on working postures such as leaning forward, twisting, bending, and occupational lifting was not available in the present study. Hence the effect of these factors was not possible to take into account in the analyses. It is also possible that the lower socioeconomic status among individuals with greater occupational physical loading may be an uncontrolled confounding factor in the present study.

## 5 CONCLUSION

In conclusion, the present study suggests a significant association between occupational physical loading and severe disc degeneration at the lower lumbar spine in postmenopausal women. Individuals in occupations with heavy physical loading may have an increased risk for work‐related disability due to more severe disc degeneration. However, the occupational loading was only self‐rated and this may lead to an overstatement of the results of the present study. These results should be taken into account in clinical practice, especially in occupational medicine and healthcare.

## AUTHOR CONTRIBUTIONS

All authors made substantial contributions to this article. J.S and S.S designed the study. S.S and H.H drafted the manuscript, contributed equally to the study, and share co‐first authorship. S.S, H.H, and J.S conducted the study. S.S, H.H, H.K, and J.S collected the data. J.S, R.S, S.S, and T.R interpreted the data. S.S revised occupational medicine aspects of the study. All authors revised the manuscript content and approved the final manuscript.

## DISCLOSURES

*Approval of the research protocol*: The study protocol was approved by the ethics committee of Kuopio University Hospital (KUH) and conformed to the Declaration of Helsinki. Ethics approval number: 1217/13.02.00/2018 *Informed Consent*: Informed written consent from the participants was collected. *Registry and the registration no*. *of the study*: N/A. *Animal studies*: N/A. *Conflict of interest*: The funding source had no role in the design, practice, or analysis of this study. All authors declare no conflict of interest for this article.

## DATA AVAILABILITY STATEMENT

Data are available upon reasonable request.

## Supporting information

Supplementary Material
