# Supplementary material for: Association between severe lumbar disc degeneration and self‐reported occupational physical loading
Source: J Occup Health. 2022 Jan 27;64(1):e12316. doi: 10.1002/1348-9585.12316 (PMC8793002; doi:10.1002/1348-9585.12316)
Supplement: Supplementary file 1 — Supplementary Material [file JOH2-64-e12316-s001.docx]

**Figure S1. L1 – S1 mean degeneration grade in different age groups according to Pfirrmann classification**

**
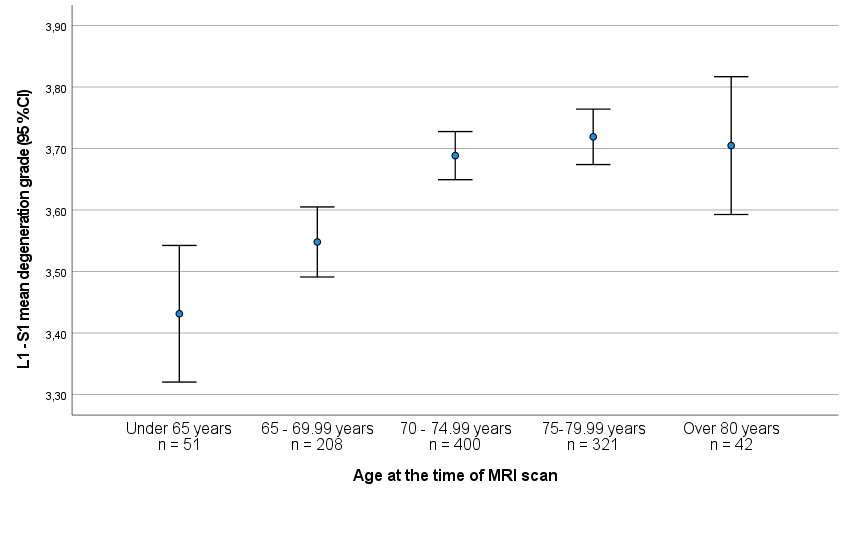
**

**p < 0.001
Adjusted p = 0.005
R-squared = 0.031
PCC = 0.177 (p < 0.001)**

- A clear trend of increasing mean degeneration grade along with age can be seen in Figure. Vertical lines represent the 95 % confidence intervals of the mean degeneration grade of all 5 lumbar vertebral levels (L1-S1) according to age groups. The increase in degeneration also slowed down with age.
- The difference in the mean degeneration grade between the different age groups was significant in ANOVA analysis (p<0.001).
- When several confounding factors including age at the time of MRI scan, BMI, time from occupational loading questionnaire to MRI scan, smoking history in years, the total number of chronic medical conditions, leisure time physical activity during the last year before the 5-year questionnaire (average hours per week), leisure time physical activity at the age of 11 to 17 years (Yes / No), and working years in the occupation were taken into account by using them as covariates in ANCOVA analysis the result remained significant (adjusted p=0.005).
- The Pearson correlation coefficient between the age group and L1-S1 mean degeneration grade was 0.177 (p<0.001).

**Table S1. Occupational classes according to the category of occupational physical loading**

**Sedentary Light Moderate Heavy Total**

Technical, Engineering 1 1 1 0 3

Chemical, Physical and
Life Science Technicians 1 2 2 0 5

Health Professionals (Medical
Doctors and Nursing Professionals) 6 23 35 21 85

Other Health Professionals 2 4 6 0 12

Teaching Professionals 3 27 12 1 43

Religious Professionals 0 0 0 1 1

Legal Professionals 1 0 0 0 1

Other Social Professionals 9 6 26 8 49

Administrators and Officials,
Public Administration 2 1 0 0 3

Business Administration 7 0 0 0 7

Accountants and Auditors 10 2 2 1 15

Secretaries and Typists 25 1 3 1 30

Other Office Workers 69 9 4 1 83

Wholesale and Retail Trade 1 3 13 5 22

Securities and Finance Dealers
and Brokers 2 1 0 0 3

Commercial Sales Representatives 0 0 1 0 1

Other Sales Workers 11 3 73 17 104

Farmers 0 0 6 17 23

Agricultural Workers 0 4 54 108 166

Fishermen and related workers 0 0 0 1 1

Mining and Mineral
Processing Plant Operators 0 0 1 0 1

Drivers, Road Transport 3 0 0 1 4

Traffic services 0 0 1 0 1

Postal-, telegraph and telephone
services 6 1 3 0 10

Letter carriers and Messengers 0 0 6 3 9

Spinners, Weavers, Knitters
and Related Workers 1 0 6 5 12

Tailors, Dressmakers 10 0 15 5 30

Metal Processing and
Finishing Plant Operators 0 0 1 1 2

Machine Shop Workers 0 1 3 0 4

Electrical and Electronic
Trades Workers 0 0 1 0 1

Carpenters, Joiners, Cabinetmakers
and Related Workers 0 0 2 5 7

Construction Trades, Other 0 0 1 2 3

Handicraft and Printing
Workers 1 0 0 2 3

Food Processing Workers 0 0 11 11 22

Chemical Process
and related Workers 0 0 2 0 2

Packers, Labellers 1 0 4 2 7

Stationary engine operators 1 0 1 0 2

Storage and warehouse workers 0 1 6 1 8

Protective Services
Workers 0 0 1 0 1

Housekeepers, Cooks and
Related Workers 0 0 57 39 96

Waiters and Bartenders 0 1 8 7 16

Building Caretakers and Cleaners 0 3 53 52 108

Barbers, Hairdressers, Beauticians
and Related workers 1 1 4 2 8

Launderers and Pressers 0 0 4 1 5

Other service occupations 1 1 1 0 3

**Total 175 (17.1 %) 96 (9.4 %) 430 (42.1 %) 321 (31.4 %) 1022**

- The table presents the distribution of the study population into different occupational classes. Information on self-rated occupational physical loading is based on OSTPRE 5-year follow-up questionnaire data.

**Table S2. Distribution of disc degeneration grades at different vertebral levels (N = 1022)**

**Pfirrmann
grade** L1-L2 L2-L3 L3-L4 L4-L5 L5-S1 Total

1 0 1 (0.1 %) 0 0 2 (0.2 %) 3 (0.1 %)

2 37 (3.6 %) 12 (1.2 %) 9 (0.9 %) 5 (0.5 %) 38 (3.7 %) 101 (2.0 %)

3 588 (57.5 %) 514 (50.3 %) 480 (47.0 %) 287 (28.1 %) 264 (25.8 %) 2133 (41.7 %)

4 347 (34.0 %) 414 (40.5 %) 465 (45.5 %) 597 (58.4 %) 457 (44.7 %) 2280 (44.6 %)

5 50 (4.9 %) 81 (7.9 %) 68 (6.7 %) 133 (13.0 %) 261 (25.5 %) 593 (11.6 %)

5110 (100 %)

- The majority of the discs (97.9 %) were within the higher Pfirrmann degeneration groups 3 to 5.
- The severity of disc degeneration was more substantial at the two lowest lumbar vertebral levels. The mean degeneration grades for each vertebral level can be found in Table 1.

**Table S3. Distribution of diagnosis or indication related to lumbar MRI and L1-S1 degeneration**

**Diagnosis L1-S1 mean deg. L1-S1 severe
 Frequency (%) grade (SD) degeneration (%)**

Spinal stenosis 464 (45.4 %) 3.73 (0.42) 142 (30.6 %)

Spondylosis without myelopathy or radiculopathy, lumbosacral region 24 (2.3 %) 3.63 (0.37) 7 (29.2 %)

Difficulty in walking 6 (0.6 %) 3.73 (0.50) 3 (50.0 %)

Radiculopathy 6 (0.6 %) 3.50 (0.47) 2 (33.3 %)

Lower limb pain or arthrosis 32 (3.1 %) 3.63 (0.46) 10 (31.3 %)

Low back pain 65 (6.4 %) 3.52 (0.37) 9 (13.8 %)

Lumbago with sciatica 46 (4.5 %) 3.62 (0.35) 13 (28.3 %)

Multiple myeloma 7 (0.7 %) 3.63 (0.31) 1 (14.3 %)

Other spondylosis with radiculopathy 5 (0.5 %) 4.0 (0.68) 3 (60.0 %)

Other intervertebral disc degeneration 43 (4.2 %) 3.62 (0.36) 5 (11.6 %)

Dorsalgia, unspecified 6 (0.6 %) 3.43 (0.15) 0 (0.0 %)

Vertebral fracture 11 (1.1 %) 3.60 (0.37) 1 (9.1 %)

Spondylolisthesis 33 (3.2 %) 3.69 (0.38) 10 (30.3 %)

Lumbar and other intervertebral disc disorders with radiculopathy 116 (11.5 %) 3.69 (0.38) 22 (19.0 %)

Rheumatic disease 14 (1.4 %) 3.50 (0.41) 3 (21.4 %)

Breast cancer 10 (1.0 %) 3.68 (0.41) 3 (30.0 %)

Spondylolysis 5 (0.5 %) 3.64 (0.26) 1 (20.0 %)

Other cancer 20 (2.0 %) 3.45 (0.37) 4 (20.0 %)

Sciatica 14 (1.4 %) 3.64 (0.41) 4 (28.6 %)

Other diagnosis 95 (9.3 %) 3.66 (0.41) 21 (22.1 %)

**Total**  **1022 (100 %)**

- Supplementary table 3 represents the distribution of diagnosis or indication related to lumbar MRI. Mean degeneration grades for the whole lumbar spine (L1-S1, five intervertebral levels) are presented for each diagnosis group. In addition, the number and proportion of study subjects classified into severe degeneration groups are presented for each diagnosis group.

**Table S4. Occupational classes from MRI-sample compared to Finnish Job Exposure Matrix (FINJEM)**

Sedentary work Sitting Heavy work Heavy
 Study sample FINJEM Study sample FINJEM FINJEM occupational code and description

Health Professionals (Medical 6/85 (7.06 %) N/A 21/85 (24.71 %) N/A
Doctors and Nursing Professionals)

Doctors 1/4 (25.00 %) 60.00 % 0/4 (0.00 %) 0.00 % 22213 Other medical doctors

Chief nursing officers 4/6 (66.67 %) 46.15 % 0/6 (0.00 %) 7.69 % 22301 Matrons

Head nurses 1/6 (16.67 %) 41.67 % 1/6 (16.67 %) 8.33 % 22302 Ward sisters

Nurses 0/30 (0.00 %) 12.94 % 3/30 (10.00 %) 38.10 % 32311 Nurses

Practical nurses 0/26 (0.00 %) 2.90 % 16/26 (61.54 %) 68.12 % 51321 Practical nurses

Practical mental nurses 0/5 (0.00 %) 18.18 % 0/5 (0.00 %) 18.18 % 5132M Practical mental and mental
 handicap nurses

Rest of the health professionals 0/8 (0.00 %) N/A 1/8 (12.50 %) N/A N/A

Other health professionals 2/12 (16.67 %) 11.11 % 0/12 (0.00 %) 0.00 % 2229 Health professionals (exept nursing)

Teaching professionals 3/43 (6.98 %) 2.86 % 1/43 (2.33 %) 0.0 % 2331 Primary education teaching prof.

Other social professionals 9/49 (18.37 %) N/A 8/49 (16.33 %) N/A N/A

Social workers 4/5 (80.00 %) 63.64 % 0/5 (0.00 %) 9.09 % 24461 Social workers

Childminders and kindergarten 0/15 (0.00 %) 2.22 % 2/15 (13.33 %) 26.67 % 51311 Childminders and kindergarten
asisstants assistants

Assistant nurses and hospital 0/18 (0.00 %) 2.44 % 6/18 (33.33 %) 43.90 % 91321 Assistant nurses and hospital ward ward assistants assistants

Other social professionals 5/11 (45.45 %) N/A 0/11 (0.00 %) N/A N/A

Accountants and auditors 10/15 (66.67 %) 100.00 % 1/15 (0.67 %) 0.00 % 2411 Accountants

Secretaries and typists 25/30 (83.33 %) 91.14 % 1/30 (3.33 %) 1.25 % 4115 Secretaries

Other office workers 69/83 (83.1 %) 72.00 % 1/83 (1.20 %) 8.00 % 4190 Other office clerks

Wholesale and Retail Trade

Small shop keepers/ retailers 1/22 (4.54 %) 6.67% 5/22 (22.72 %) 26.67 % 34192 Shop managers and small
 shopkeepers

Other sales workers 11/104 (10.58 %) 20.21 % 17/104 (16.35 %) 31.58 % 52202 Salespersons and cashiers

Farmers 0/23 (0.00 %) 0.00 % 17/23 (73.91 %) 90.20 % 6130 Crop and animal producers and workers

Agricultural workers 0/166 (0.00 %) N/A 108/166 (65.06 %) N/A 6130 Crop and animal producers and workers

Farmer’s wife 0/149 (0.00 %) N/A 96/149 (64.43 %) N/A

Other agricultural worker 0/1 (0.00 %) 0.00 % 1/1 (100 %) 90.91 % 6123 Farmer’s locums

Gardener/Park worker 0/16 (0.00 %) 0.00 % 11/16 (68.75 %) 62.50 % 6112M: Gardeners, horticultural and nursery growers

Postal-, telegraph and telephone 6/10 (60.00 %) 0/10 (0.00 %)
services

Postal Clerks 1/5 (20.00 %) 43.75 % 2/5 (40.00 %) 18.75 % 3419M Superiors at banks and post offices,
 service station and other managers

Telephone switchboard operators 5/5 (100 %) 79.49 % 0/5 (0.00 %) 2.56 % 4223 Telephone switchboard operators and
 emergency workers

Spinners, Weawers, Knitters and 1/12 (8.33 %) 23.81 % 5/12 (41.67 %) 52.38 % 826C1: Weawing-, knitting- and sewing-
related workers machine operators

Tailors, dressmakers 10/30 (33.33 %) 35.71 % 5/30 (16.67 %) 57.14 % 826C2 Sewing, bleaching, dyeing- and
 cleaning-machine operators

Food processing workers 0/22 (0.00 %) 46.15 % 11/22 (50.00 %) 30.77 % 741 Food processing and related trades
 workers

Housekeepers, cooks and related 0/96 (0.00 %) 0.00 % 39/96 (40.62 %) 46.51 % 5122 Cooks
workers

Waiters and Bartenders 0/16 (0.00 %) 4.65 % 7/16 (43.75 %) 39.53 % 5123 Waiters, waitresses and bartenders

Building caretakers and cleaners 0/108 (0.00 %) 0.00 % 52/108 (48.14 %) 54.12 % 91322 Cleaners

- Supplementary Table 4 represents the largest occupational groups presented in Supplementary Table 1. The proportion of heavy work and sedentary work for the study population are presented in the Table. Data from Finnish Job Exposure Matrix (FINJEM) was used in comparison to the study population. FINJEM-database provides information on how big proportion of the workers in different occupations are exposed to specific work-load factors. Some occupational groups in the study sample were too wide and they were split into more specific subgroups.
- FINJEM-database is available online: https://tyoelamatieto.fi/en/dashboards/physical-exposure
